# Chronic hypersensitivity pneumonitis in the southeastern United States: an assessment of how clinicians reached the diagnosis

**DOI:** 10.1186/s12890-020-1072-7

**Published:** 2020-02-05

**Authors:** Jessie P. Gu, Chen-Liang Tsai, Nicholas G. Wysham, Yuh-Chin T. Huang

**Affiliations:** 1 Department of Medicine, Duke University Medical Center, 1821 Hillandale Road, Suite 25A, Durham, North Carolina 27705 USA; 2 Department of Chest Medicine, Tri-service General Hospital, Taipei, Taiwan

**Keywords:** Chronic hypersensitivity pneumonitis, Diagnosis, Exposure

## Abstract

**Background:**

Chronic hypersensitivity pneumonitis (cHP) is a disease caused by exposure to inhaled environmental antigens. Diagnosis of cHP is influenced by the awareness of the disease prevalence, which varies significantly in different regions, and how clinicians utilize relevant clinical information. We conducted a retrospective study to evaluate how clinicians in the Southeast United States, where the climate is humid favoring mold growth, diagnosed cHP using items identified in the international modified Delphi survey of experts, i.e., environmental exposure, CT imaging and lung pathology,

**Methods:**

We searched Duke University Medical Center database for patients over the age of 18 with a diagnosis of cHP (ICD-9 code: 495) between Jan. 1, 2008 to Dec. 31, 2013 using a query tool, Duke Enterprise Data Unified Content Explorer (DEDUCE).

**Results:**

Five hundred patients were identified and 261 patients had cHP confirmed in clinic notes by a pulmonologist or an allergist. About half of the patients lived in the Research Triangle area where our medical center is located, giving an estimated prevalence rate of 6.5 per 100,000 persons. An exposure source was mentioned in 69.3% of the patient. The most common exposure sources were environmental molds (43.1%) and birds (26.0%). We used Venn diagram to evaluate how the patients met the three most common cHP diagnostic criteria: evidence of environmental exposures (history or precipitin) (E), chest CT imaging (C) and pathology from lung biopsies (P). Eighteen patients (6.9%) met none of three criteria. Of the remaining 243 patients, 135 patients (55.6%) had one (E 35.0%, C 3.3%, P 17.3%), 81 patients (33.3%) had two (E + C 12.3%, E + P 17.3%, C + P 4.9%), and 27 patients (11.1%) had all three criteria (E + C + P). Overall, 49.4% of patients had pathology from lung biopsy compared to 31.6% with CT scan.

**Conclusions:**

Environmental mold was the most common exposure for cHP in the Southeast United States. Lung pathology was available in more than half of cHP cases in our tertiary care center, perhaps reflecting the complexity of referrals. Differences in exposure sources and referral patterns should be considered in devising future diagnostic pathways or guidelines for cHP.

## Background

Chronic hypersensitivity pneumonitis (cHP) is an immunologically mediated lung disease caused by a persistent or repeated exposure to inhaled environmental or occupational antigens resulting in bronchoalveolar inflammation and progressive fibrosis [1, 2]. While previously characterized as acute, subacute, and chronic, recent literature has transitioned to classifying HP as acute or inflammatory and chronic or fibrotic forms as these two forms have been associated with differences in survival [3]. Failure to recognize cHP early and identify an inciting antigen to avoid can potentially lead to permanent pulmonary disability and even death due to progressive respiratory failure [4]. Therefore, it is crucial to make an accurate clinical diagnosis of cHP and distinguish from other types of interstitial lung disease (ILD) as treatment is very different and prognosis of nonfibrotic cHP is generally better than idiopathic pulmonary fibrosis [5, 6].

cHP can be difficult to diagnose as there are no unique diagnostic features. Although the diagnosis may be suggested by a combination of information about exposure obtained from clinical history or laboratory tests, radiologic findings on chest CT imaging and sometimes pathology, uncertainty of exposure history, low sensitivity of serological tests, as well as overlapping histopathologic and radiologic features with other forms of ILD may affect the diagnostic confidence of clinicians [7, 8]. Morisset et al. recently used a modified Delphi survey from multidisciplinary experts and identified diagnostic items considered to have the highest level of importance. These items included identification of a causative antigen, time relation between exposure and disease, mosaic attenuation on CT imaging, and poorly formed non-necrotizing granulomas on pathology [9].

Clinicians’ approach to diagnosing cHP may also be affected by their perception of the prevalence of the disease, which has significant geographic variations depending on the prevalence of inciting environmental exposures [10]. Several prior reports in the medical literature described cohorts of patients with hypersensitivity pneumonitis in different geographic areas. One from Japan described a cohort of 222 patients over 10 years (2000–2009) using a nationally administered questionnaire survey and determined that bird-related HP was more common than summer-type HP which was previously thought to be the most prevalent variant in 1999 [11]. Another study from Denver, Colorado described a cohort of 142 chronic HP confirmed by surgical lung biopsy over 27 years (1982–2008) and showed the most common exposure was among pigeon breeders and bird fanciers [4]. Additional cohort studies conducted in Belgium and Spain also showed the most common inciting antigen to be bird-related [12, 13]. These studies have limitations of their generalizability as the prevalence of bird exposure and exposure to other sources of cHP are influenced by cultural, occupational and socioeconomic factors as well as local climate, which vary in different countries and regions.

In the Southeast United States, the weather tends to be warm and humid. Such climate as well as its proximity to Atlantic Ocean promotes mold growth, a major exposure culprit for cHP [14, 15]. Therefore we conducted a retrospective study on data obtained from 2008 to 2013 to evaluate how clinicians diagnosed cHP in this region using items that were given the highest level of importance by the experts in the international modified Delphi survey, i.e., environmental exposure, CT imaging and lung pathology, that was published in 2017 [16].

## Methods

### Patient selection

Patients over the age of 18 diagnosed with cHP (ICD-9 code: 495) between Jan. 1, 2008 to Dec. 31, 2013 were identified from the database at Duke University Medical Center using the patient query tool, Duke Enterprise Data Unified Content Explorer (DEDUCE). We reviewed their medical records and retained those patients whose diagnosis was thoroughly annotated and confirmed by a pulmonologist or an allergist. We recorded the following data of the patients: demographics, smoking history, zip code, occupational or environmental history, pulmonary function, CT scan, and histopathology (if available). The Duke Health institutional review board approved this study (IRB number Pro00052804).

### Diagnostic criteria

We used the following three criteria to evaluate how clinicians made the diagnosis of cHP: evidence of environmental or occupational exposures (E); chest CT imaging (C), and pathology from lung biopsies obtained by bronchoscopy or video-assisted thoracoscopic surgery (VATS) (P). Positive evidence of environmental or occupational exposure included a known cause of cHP, exposure preceded the onset of symptoms, improvement in symptoms after withdrawal of exposure, and/or the presence of serum precipitating antibodies (Hypersensitivity Pneumonitis FEIA Panel II, Mayo Clinic Laboratories; Hypersensitivity Pneumonitis Panel, Test Code: 401749P, Viracor). Findings on CT scan that supported the diagnosis of cHP included characteristic air-trapping or mosaic attenuation, along with other radiologic findings such as upper lobe predominance, reticular or ground glass opacities, bronchiectasis, centrilobular nodules and fibrosis, and/or cHP was mentioned in the differential diagnosis described in radiology report. Pathology findings are considered supportive of cHP if poorly formed granulomas and/or organizing pneumonia in bronchiolocentric distribution were present and were felt to be consistent with cHP, or other pathological findings were present and cHP was mentioned in the differential diagnosis.

### Statistical analysis

Descriptive statistics were performed using Excel (Microsoft, Seattle, WA). Normally distributed variables including age and pulmonary function test measurements were presented as mean and standard deviation (SD). Categorical variables including race and type of exposure were expressed as the number of patients and the percentage of patients for each characteristic out of the total number of patients included in the study. The Venn diagram was created using a free online program Meta-chart (https://www.meta-chart.com/). The geographic mapping of patients included in the study was created using geocoding software in DEDUCE.

## Results

Five hundred patients were initially identified using the 495 ICD-9 code. After reviewing medical records, we narrowed the study population to 261 patients (52.2%) whose diagnosis was thoroughly annotated by a pulmonologist or an allergist. The clinical characteristics of these 261 patients were summarized in Table 1. The mean age was 57 ± 14 years and 157 patients were female (60.1%). More than half of the patients (54.4%) were non-smokers. Pulmonary function in general showed mild restriction with a reduction in DLCO. An inciting agent was identified in 181 patients (69.3%) while in the other 80 patients (30.7%), the inciting agent was unknown. In patients with known inciting agents, the most common exposure identified was environmental molds (43.1%). Birds (e.g. cockatiels, pigeons) were the second most common exposure source (26.0%).

**Table 1:** Clinical and Physiologic Characteristics in Patients with HP

Characteristics	All patients (*N* = 261)
Demographics	
Age of diagnosis, y	57 ± 14
Female sex	157 (60.1)
Race	
Caucasian/White	217 (83.1)
African American/Black	29 (11.1)
American Indian	1 (0.3)
Multiracial	1 (0.3)
Other	6 (2.3)
Not reported	7 (2.7)
Cigarette smoking status	
Nonsmoker	142 (54.4)
Former/Current Smoker	94 (36.0)
Not reported	23 (8.8)
Exposure	
Identified IA	181 (69.3)
Unidentified IA	80 (30.7)
Type of Exposure	
Mold	78 (43.1)
Bird	47 (26)
Hot tub	3 (1.7)
Dust	14 (7.7)
Farmer’s lung	13 (7.2)
Occupational	10 (5.5)
Drugs	11 (6.1)
Positive ANA	50 (19.2)
Pulmonary function tests, % predicted	
TLC	71 ± 21
RV	73 ± 36
FVC	68 ± 22
FEV_1_	67 ± 22
FEV_1_/FVC	78 ± 10
D_LCO_	60 ± 25
CT findings	89 (34.1)
Mosaic attenuation	39 (14.9)
Centrilobular nodules	16 (6.1)
Bronchiectasis	38 (14.6)
Fibrosis	11 (4.2)
Nonspecific imaging	12 (4.6)
Biopsy obtained	120 (49.4)
VATS	92 (76.7)
Classical TBB	13 (10.8)
Both	15 (12.5)

Values are given as the mean ± SD or No. (%). *HP* Hypersensitivity pneumonitis, *IA* Inciting antigen, *TLC* Total lung capacity, *RV* Residual volume, *FVC* Forced vital capacity, *FEV*_*1*_ Forced expiratory volume in one second, *D*_*LCO*_ Diffusing capacity of lung for carbon monoxide, *VATS* Video assisted thoracoscopic surgery, *TBB* Trans-bronchial biopsy. Nonspecific imaging included scattered ground glass opacity (GGO), peripheral consolidation, interstitial infiltrate, transient GGO

**Fig. 1:**
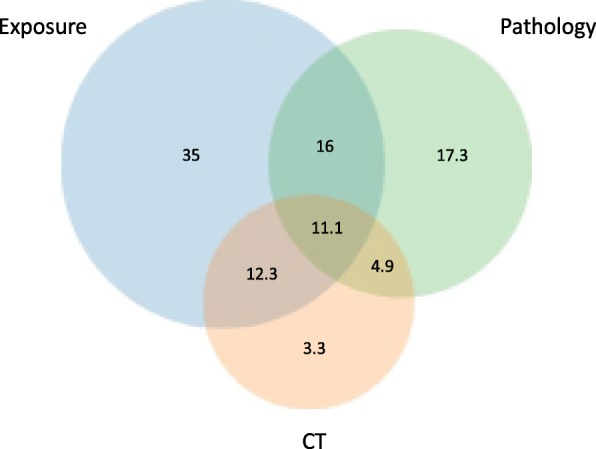
Venn diagram demonstrating the percentages of patients diagnosed by exposure

Eighteen patients did not have any of the three criteria for cHP (6.9%). The criteria used by the clinicians to reach the diagnosis in these 18 patients are summarized in Table 2. The clinician assessment of steroid responsiveness and non-characteristic CT findings were major factors. Venn diagram analysis on the remaining 243 patients showed 135 patients (55.6%) with one criterion: E 85 (35.0%), C 8 (3.3%), P 42 (17.3%); 81 patients (33.3%) with two criteria: E + C 30 (12.3%), E + P 39 (16.0%), C + P 12 (4.9%); and 27 patients (11.1%%) with all three criteria (E + C + P) (Fig. 1). Overall, 50.6% of patients had pathology from lung biopsy compared to 31.6% with CT scan.

**Table 2:** Diagnostic characteristics of patients diagnosed with cHP but did not meet the three criteria used in this study

Diagnostic approach	Number of patients = 18 (%)
Steroid responsiveness	9 (50)
Nonspecific imaging (scattered GGO, peripheral consolidation, interstitial infiltrate, transient GGO)	12 (66.7)
Eosinophilia	3 (16.7)

**Fig. 2:**
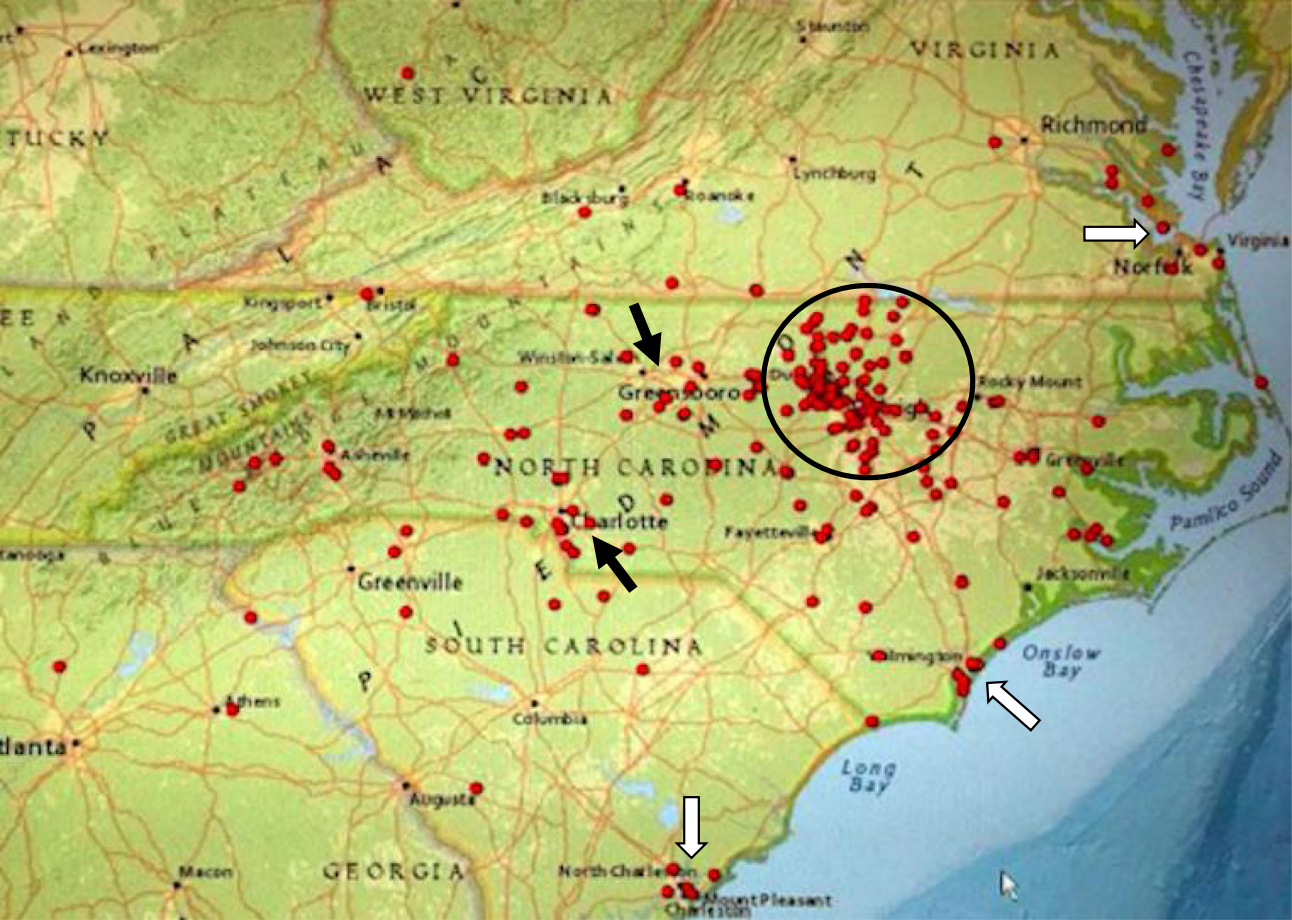
Map of the Carolinas and southern Virginia that shows the distribution of 238 cases of cHP who had a physical address that could be verified. The map was generated by the DEDUCE-Geo software. DEDUCE-Geo uses both ArcGIS Server (Esri, Redlands, CA) and JavaScript to execute the geospatial visualization of a cohort defined within DEDUCE. Each red dot represents one case of cHP. There is a major cluster around the Research Triangle area (circle). There also seemed to have more cases in other larger cities, such as Greensboro and Charlotte (black arrows) and in coast regions, such as Norfolk VA, Wilmington NC and Charleston SC (white arrows)

Geographic distribution of patients whose addresses were able to be mapped (*n* = 243) was created using the geocoding software in DEDUCE. As expected, most patients were from the Carolinas, Virginia and neighboring states. A distribution map of the Carolinas and southern Virginia is shown in Fig. 2. This map shows larger clusters of patients in and near the Research Triangle area where our medical center is located, in other larger cities, such as Greensboro and Charlotte and along the coast from Norfolk VA, Wilmington NC to Charleston SC.

Among the 239 patients whose initial HP diagnosis was not confirmed, 29% had no underlying lung disease diagnosed. In the remainder of the patients, asthma was the most common diagnosis (18.5%), followed by non-HP ILD (16.5%), COPD (12.5%) and pneumonia (7.5%) (Table 3).

**Table 3:** Underlying pulmonary diagnosis among patients misdiagnosed with cHP

Diagnosis	Number of patients = 200 (%)
COPD	25 (12.5)
Asthma	37 (18.5)
ILD	33 (16.5)
Connective tissue disease	5 (2.5)
Pneumonia	15 (7.5)
Cancer	7 (3.5)
Sarcoid	5 (2.5)
No lung diagnosis	58 (29)

## Discussion

Our study investigated how clinicians diagnosed cHP during a period when specific diagnostic guidelines had not been published. Some of the recommendations in the guidelines, such as multidisciplinary discussion, bronchoscopy with BAL lymphocytosis and cryobiopsy, thus were not available to the clinicians. Therefore, we included only cHP diagnosed by specialty physicians, i.e., pulmonologists and allergist/immunologists, who are more familiar with cHP than general practitioners. We then used the top three items in the Delphi survey to determine retrospectively how these three items were used to diagnosed cHP [16].

Note that our study was performed in a tertiary medical center in Southeast United States, a region near Atlantic Ocean with high humidity favoring mold growth. The prevalence of cHP in this region is unknown, but the pretest probability is high. The prevalence of cHP varies considerably depending upon case definitions, intensity of exposure to inciting antigens, climate condition, local practice patterns and host risk factors [17–20]. Using a large insurance database (150 million subjects), Fernández Pérez et al. estimated the one-year prevalence rate of cHP ranged from 1.67 to 2.71 per 100,000 persons [19]. In our study, more than half of the 261 patients were in the Research Triangle area that had an average population of approximately 2 million during the study period (2008–2013). This would give a prevalence rate of about 6.5 per 100,000 persons. The higher humidity in the southeast regions of the United States that promotes mold growth is likely an important factor because the main exposure source in our cohort was environmental molds. The 261 cases over 6 years in our study (43.5/year) also represented a greater case encounter than that of the reported cohorts. For example, the Japanese cohort that was compiled by questionnaire had 222 patients over 10 years (22.2/year) [11]. The Denver cohort that included only patients with pathology had 142 patients over 27 years (5.3/year) [4]. The higher case encounter rate in our medical center could be in part due to the referral bias although the higher prevalence of cHP is also contributory. Clinicians practicing in regions that have high prevalence of environmental mold growth should have high suspicion of cHP when evaluating patients with interstitial lung diseases.

Our study found that the most common criterion used by the clinicians in the diagnosis of cHP was environmental exposure (74.5% of the cohort). The importance of exposure history in the diagnosis of cHP has been repeatedly demonstrated in the literature [16, 21, 22]. In the studies by Johannson et al. and Salisbury et al., exposure history was one of the two most common criteria for the diagnosis of cHP [21, 22]. A multitude of causative agents can be found in the workplace and home environment [21]. Identifying a clear inciting antigen based on clinical history with a definitive timeline of exposure preceding symptoms helps the clinicians diagnose cHP. Removal of the exposure is also considered the cornerstone of cHP diagnosis [23]. When the exposure source can be pinpointed, such as birds, hay, antigen avoidance by removal from exposure or wearing protective devices tends to be more effective in halting or reversing the disease progression [4]. An extensive search, however, may not reveal a clear source because the latency between exposure and onset of disease varies widely from months to years and occult or low-level persistent exposure to unknown source makes it challenging to discern the type of antigen [23]. If inciting agents cannot be identified by clinical history or laboratory tests, like in many cases of fibrotic HP, the diagnostic confidence may decrease and clinicians may more likely resort to lung biopsy. The inability to identify an inciting antigen was independently associated with shortened survival even after controlling for important variables such as the presence of pulmonary fibrosis (4).

The most characteristic features are air-trapping or the mosaic attenuation predominantly in the upper lobes. There may also be non-specific findings including airway-centric disease, centrilobular nodules and ground glass opacities. Imaging may also overlap with radiologic features of other ILD such as linear densities, honeycombing that make it challenging for clinicians to diagnose cHP [24–26]. It is important to distinguish cHP from other ILD, such as idiopathic pulmonary fibrosis IPF as the non-fibrotic HP is reported to have a better prognosis [8, 27]. Having a more defined pattern for radiologists to discern cHP from other forms of ILD may also enhance the specificity of HRCT. Salisbury et al. derived and validated a diagnostic model for cHP based solely on radiologic findings when the extent of mosaic attenuation or air trapping is greater than reticulation and the disease has diffuse axial distribution with a specificity < 90% [28].

CT scan is considered the most useful non-invasive tool in the diagnosis of cHP [21, 22]. The most characteristic features are air-trapping or the mosaic attenuation predominantly in the upper lobes. There may also be non-specific findings including airway-centric disease, centrilobular nodules and ground glass opacities. Imaging may also overlap with radiologic features of other ILD such as linear densities, honeycombing that make it challenging for clinicians to diagnose cHP [24–26]. It should be noted that not all patients had CT reports in our system when our specialists made the diagnosis of HP. Conceivably most if not all patients should have CT chest reports before the referral. The specialists may have seen them, but we did not have access to these outside reports.

In our study, supportive CT findings were only present in 31.6% of the patients, and almost half of these patients also had undergone biopsy. The majority of these patients (92) had VATS, 13 patients had bronchoscopy and 15 patients had both (Table 1). In the Delphi survey, bronchoscopy with bronchoalveolar lavage (BAL) and transbronchial biopsy was recommended to increase diagnostic confidence [16]. BAL as the diagnostic test is poorly characterized in patients with fibrotic cHP and a wide range conditions can be associated with lymphocytosis [29–31]. Adams et al. also demonstrated the combination of transbronchial biopsy and BAL increases the likelihood that the procedure will give adequate information to allow a confident diagnosis of cHP as well as possibly reducing the need for more invasive surgical lung biopsy [31]. However, this study also showed that the diagnostic yield of BAL was low [31]. Therefore, while not diagnostic on its own, BAL can be helpful in the diagnosis of HP when combined with other clinical findings. Note that there is regional variability in the use of lymphocytosis in BAL in the diagnosis of cHP. During the study period, clinicians in our institution did not rely on BAL or transbronchial biopsy to diagnose cHP. The findings in our study that pathology from VATS biopsy was used more frequently than bronchoscopy to diagnose cHP reflected this practice pattern. More robust bronchoscopy data with better discriminant power, e.g., those from cryobiopsy, would help decrease regional variations in clinical practice. Publication of diagnostic guidelines may also make more clinicians aware of the utility of BAL lymphocytosis.

It is also notable that 6.9% of patients who were given the diagnosis of cHP did not have any of the three criteria. Among these 18 patients, 50% were diagnosed based on responsiveness to steroids, 66.7% were diagnosed based on nonspecific imaging findings without mosaic attenuation, and 16.7% were diagnosed based on eosinophilia (Table 2). The first two items (steroid responsiveness and nonspecific imaging) did not meet consensus in the modified Delphi survey [16]. Allergic manifestations, such as wheezes, reached unimportant threshold [16]. Another finding in our study was almost 50% of the patients who carried an ICD-9 code of cHP (495) actually did not have cHP as evaluated by pulmonary or allergy specialists. Among these patients, 29% had no underlying lung disease. For those patients who did have lung disease, asthma is most common (18.5%) followed by non-HP ILD (16.5%), COPD (12.5%) and pneumonia (7.5%) (Table 3). The causes for the unusually high percentage of miscoding are unclear but may be related in part to the unfamiliarity of clinicians in the diagnostic criteria of cHP and/or ICD coding [32, 33]. It is also possible that HP entered initially by the referring physicians remained on the problem list of our electronic health record system even after the HP diagnosis was later refuted. If HP was not deleted from the problem list, it would show up during the search.

## Conclusion

Our study is the first to characterize cHP in the warm and humid Southeast United States where the prevalence of HP appeared to be higher than prior studies in less humid regions. Exposure history remained the most common diagnostic criterion used to diagnose cHP by the clinicians. The most common exposure was environmental molds, unlike other cohort studies in which birds were primary culprits. More lung biopsies were pursued for the diagnosis in our patient cohort probably reflecting the complex nature of referral in a major tertiary medical center. These results underscore the importance that regional variations in disease prevalence and clinical practice patterns be considered in devising future diagnostic pathways for cHP.

## Data Availability

The datasets used and/or analysed during the current study are available from the corresponding author on reasonable request.

## Abbreviations

BAL: Bronchoalveolar lavage
C: CT
cHP: Chronic hypersensitivity pneumonitis
COPD: Chronic obstructive pulmonary disease
DEDUCE: Duke Enterprise Data Unified Content Explorer
DLCO: Diffusing capacity of lung for carbon monoxide
E: Exposure
ILD: Interstitial lung disease
P: Pathology
VATS: Video assisted thoracoscopic surgery
